# Research on Literature Clustering Algorithm for Massive Scientific and Technical Literature Query Service

**DOI:** 10.1155/2022/3392489

**Published:** 2022-08-21

**Authors:** Chen Zhang

**Affiliations:** Wuhan University of Science & Technology Library, Wuhan 430081, Hubei, China

## Abstract

Traditional science and technology literature search mainly provides users with reliable and detailed information materials and services through technical means, data resources, and service strategies. With the development of network technology, computer technology, and information technology, digital information resources are increasing day by day, which continuously impact the traditional knowledge service mode. Some traditional technical methods and service means can no longer meet the information needs of users under large data sets. This paper proposes a model of large-scale literature search service in the context of big data by studying the technical means and service modes used for scientific and technical literature search in universities in the era of big data. Specifically, this paper proposes a method for fast literature retrieval by combining R-tree indexing for the characteristics of diverse data types and large data volume of science and technology literature. The method uses an improved k-mean clustering algorithm to construct an R-tree clustering model and improve the retrieval efficiency of the system by retrieving scientific and technical literature data through R-tree indexing. Experiments on university science and technology literature datasets show that the method in this paper improves both efficiency and precision when searching literature.

## 1. Introduction

Science and technology innovation capability is a decisive factor in the development of national science and technology undertakings and is the core of national competitiveness and an important foundation for strengthening the country and enriching the people. As an important carrier for the dissemination of scientific and technological knowledge, science and technology literature resources, which are the results of scientific and technological innovation activities, are also the basic source and important support to further enhance the scientific and technological innovation capacity and have become one of the valuable strategic resources of the country [1].

In the process of scientific and technological innovation, it is particularly important for the innovation subject to understand the history and trends of the development of the discipline, which means effective academic communication with peers [2]. And reading domain-related scientific and technical literature is the best way to realize this process. However, the prerequisite is whether the field-related literature is comprehensive in coverage and closely related in content, which is also a key factor affecting the effectiveness of innovation. Therefore, it is of great significance and demand to support the research on the acquisition methods of scientific and technical documents by innovative subjects. The existing methods of acquiring scientific and technical documents by innovative subjects are mainly based on “active search” through the search tool based on “keyword” method, but this method is influenced by human factors of searchers and the search tool, and it is often difficult to meet the requirements of document coverage and accuracy. However, this method is influenced by the human factor of the searcher and the search tool, and it is often difficult to meet the requirements of literature coverage and accuracy.

Scientific and technical documents are scientific and technical journals, papers, patents, standards, and network scientific and technical information resources in the form of text. It is huge, diverse, isolated, dynamic, heterogeneous, diverse, and complex and has strong professional, academic, and unstructured characteristics. The existing “active search” method not only requires the searcher to master the search tool, but also requires him/her to set the “keywords” accurately. However, the setting of “keywords” is greatly influenced by the searcher's scientific knowledge, personal experience, professional background, and occasional subjective factors, which not only makes the search process time-consuming and labor-intensive, but also often results in a large number of redundant and useless documents.

In the traditional manual literature search, the citation and retrieval of scientific and technical documents are done manually. With the increase of electronic literature, manual processing of literature has become overwhelming. In order to automate the processing of a large amount of literature, it is necessary to use computers to process the literature from citation to retrieval, so that the quantity and speed of literature processing are greatly increased. The development of information retrieval systems has made great progress over the decades, and many information retrieval systems have been implemented. From systems based on simple and well-used Boolean models to retrieval systems based on vector space models and probabilistic models, etc., the accuracy of information retrieval has been continuously improved [3]. However, different search systems will show different performance in the face of different literature. Chinese scientific and technical literature has its own characteristics compared with other literature: the use of standardized words; articles published in different levels of journals generally have different degrees of relevance to the searcher, and the higher the level of the journal, the higher its degree of relevance; the structure of scientific and technical literature is also relatively standard, with a clear structure, etc.

The cross-development and integration of computer science and technology with various disciplines in the information age has promoted the continuous development of information acquisition, information analysis, data processing, and knowledge management. For example, the rise of the Internet of Things, web mining, and data mining has continuously impacted the traditional technical means of information acquisition [4]. Knowledge services in the context of big data era are closely related to data mining, clustering analysis, and collaborative processing. In the research fields of text clustering algorithms, knowledge search engines, and knowledge service models, data mining and web mining related to the information knowledge acquisition means are also very much researched. Knowledge acquisition channels and approaches based on big data sets have also become hot spots and major research directions in recent years, mainly focusing on the application and practice of combining big data with cloud computing, intelligent computing, data mining, cluster analysis, and so on. However, the research, practice, and application of these technologies in the field of library knowledge services are few and far between. Thus, it can be seen that research on data mining, cluster analysis, and search engines for library knowledge services based on the background of big data can fill in or supplement the research or deficiencies in this field.

Data released from the U.S. Internet Data Center shows that the data on the Internet grows at a rate of 50% per year, and the data will double every two years, and it is noteworthy that 90% of the data volume in the Internet is generated in the past six years [5]. The main contribution of this paper is that, with the continuous growth of digital information resources and the widespread use of various intelligent terminals based on information services, the traditional knowledge retrieval model is no longer suitable for the knowledge needs of users in the big data environment. With the background of the big data era, this paper starts from the query system and studies the core element “database” that constitutes the retrieval system. The technical means and realization methods in the process of data collection, processing, and handling are analyzed, and the knowledge service model based on the background of big data is constructed by combining the clustering methods in big data. Specifically, an information retrieval method based on R-tree indexing is proposed for the problems of large quantity and variety of scientific and technical documents in the database, slow query speed, and low accuracy rate. We analyze how R-tree is constructed and propose an R-tree model that draws on the existing k-means clustering algorithm to dynamically determine the clustering centers to achieve efficient querying of scientific and technical literature data and improve the system retrieval rate through R-tree indexing.

## 2. Related Works

### 2.1. Status of Research on Large-Scale Scientific and Technical Literature Search Technology

Different kinds of documents have different logical structures; for example, legal documents are generally composed of legal texts, etc., while scientific and technical documents are composed of five parts: title, abstract, keywords, body, and references, all of which reflect the topic of the document in a macroscopic and complete way, and all five parts can be used to identify a document [6]. In this paper, the title, abstract, keywords, body, and references of scientific and technical literature are first represented as five position vectors and then synthesized into a literature vector.

Three main information retrieval models are described in detail in the literature [7]: Boolean model, vector space model, and probabilistic model. Usually, the scientific and technical literature is split into five parts, each of which forms a location space, and the whole literature space is obtained by merging the five location spaces, and the literature vectors in the literature space are calculated from the five location spaces. In this way, the importance of the words in each part's position can be highlighted. The word weights in the literature vector are based on the frequency of occurrence of words, and the most famous method for calculating word weights is the inverse literature frequency method [8], while in order to eliminate the influence of literature length on word frequency, they are all normalized, mainly the following: maximization specification, logarithmic specification, cosine normalization, and axis specification. The most commonly used methods for evaluating retrieval systems, in addition to the spatial and temporal complexity of the system, are the completeness and accuracy rates, but the completeness and accuracy rates are either in the relevant set or in the irrelevant set for a document and are binary [9].

From the query results of international top academic journals such as Nature and Science, in recent years, foreign scholars mainly focus their attention on cloud computing technology, parallel processing technology, network information analysis and performance computing, large-scale data clustering, and other directions, mainly researching graph clustering algorithms in the context of big data, data mining in the era of big data, information services, and other related technical issues [10]. It also researches information security, retrieval mode, and service strategy in the context of big data, and the core contents, related technology implementation, and information service researched are yet to be introduced to the knowledge service field of university libraries. The relevant research on knowledge service of scientific and technological literature first appeared in the information service in the business field in foreign countries, and it is believed that knowledge service is the integration of knowledge management, knowledge organization, and knowledge market, which will become an emerging industry. The main research contents are the knowledge service model based on users' information demand, the model based on information retrieval service, and knowledge sharing strategy [11]. In the course of the research, although the development of software regarding the reference consulting service model of digital libraries, the construction of information resource databases in knowledge services, and data mining and user demand analysis emerged, they were all carried out based on the traditional information service model.

To sum up, foreign information institutions and research scholars have made certain achievements in the practice and research of both big data and scientific and technical documentation services and have certain theoretical research bases. However, whether the research is based on big data or knowledge service, foreign research institutions and scholars are relatively independent in classifying these two and have not yet ventured into the intersection between knowledge service and big data for practice. It can be seen that the model of scientific and technical literature service and the realization of knowledge acquisition based on the background of big data have not been carried out in foreign countries yet. According to the degree of intelligence and semanticization of the system, the existing semantic retrieval systems for scientific and technical documents are classified into four types: semantic query extended retrieval systems, concept- or entity-centered retrieval systems, relationship-centered retrieval systems, and knowledge discovery-oriented retrieval systems [12]. These four types of retrieval systems have different degrees of semantic processing of the text of scientific and technical documents, and different degrees of intelligence and semanticization of the retrieval systems.

Semantic query expansion retrieval systems process search terms based on traditional keyword search and expand them using controlled word lists and ontologies. PubMed [13] supports MeSH-based query expansion and also PubMed queries using synonyms from UMLS [14]. QuExT [15] performs concept-oriented query expansion, and search results are ranked according to different weights assigned to concept categories by the user in advance. GO2PUB [16] performs semantic expansion of PubMed queries using semantic inheritance between terms in gene ontologies, and gene names, symbols, and synonyms are submitted to the query processor as additional keywords.

Concept- or entity-centered retrieval systems use ontologies, subject headings, and narrative lists to semantically annotate scientific and technical literature to identify knowledge in the literature, and the retrieval process is performed by matching user queries with semantic annotation results, which allows retrieval systems to use the annotation information to query for more precise results. GoPubMed [17] is the most typical of such systems, which uses gene ontologies, and it uses the gene ontology and MeSH to cite PubMed literature and is used for the structured presentation of search results, allowing users to see the main biomedical concepts relevant to the query. Compared with PubMed, GoPubMed can find relevant search results faster.

Relationship-centric retrieval systems can provide relationship-based retrieval services by discovering relationships between concepts or entities from scientific and technical literature through text mining techniques. Quertle [18] is a relationship-driven biomedical literature retrieval tool that uses a semantic-based natural language processing approach to extract subject-predicate object relationships from biomedical literature collections and discover general or specific relationships between biomedical entities (e.g., diseases and genes). And it finds general or specific relationships between biomedical entities (e.g., diseases, genes, and drugs). Using “caffeine migraine” as a search term, Quertle finds a relationship between two search terms such as “caffeine for migraine,” rather than the usual PubMed search that returns both “caffeine” and “migraine.”

CoPub 5.0 [19] developed a new technology called CoPub Discovery based on CoPub cooccurrence relationship mining, which can be used to mine indirect relationships from the literature to study the mechanisms behind diseases, link genes and pathways, and discover novel applications of existing drugs. The new technique, called CoPub Discovery, was developed based on CoPub cooccurrence mining. CoPub 5.0 provides three modes of analysis: “term search” mode to retrieve abstracts and term relationships for a term, “pair search” mode to analyze known or new relationships between term pairs, and “FACTA++” [20] finds and visualizes indirect associations between biomedical concepts such as genes, diseases, and compounds from MEDLINE abstracts, uses machine learning models to discover biomolecular events in the text, and uses cooccurrence relationships between concepts to statistically mine information. The cooccurrence relationship between concepts is used to count the hidden associations of information mining.

### 2.2. Status of Research on Text Clustering Techniques

Text clustering is a classical data mining technique, which is one of the important means to organize, summarize, and navigate textual information. Text clustering is a method of mining the content contained in text document resources by dividing them into several categories according to certain similarity criteria, so that the similarity of each category of documents can reach a predetermined standard, giving a certain information description for each category. The criteria for clustering by text clustering algorithms are high similarity between documents contained in the same category and low similarity between documents contained in different categories [21]. As an unsupervised machine learning method, it is not necessary to train the text contained in the data collection in advance or to manually label the text contained in the data collection between the processing of the data collection using this method, and the final clustering results are determined by the clustering algorithm in an unsupervised state, so it can be determined that the clustering algorithm has a certain flexibility. Therefore, it can be determined that the clustering algorithm has a certain degree of flexibility and a high automatic processing capability. At present, there are many kinds of text clustering algorithms, which are mainly classified into the following four categories according to the classification ideas of clustering algorithms.

Regarding division-based clustering algorithm for a given data set containing *n* data objects, the result of clustering using the division-based approach is to divide the data set into *k* clusters, where *k* is predetermined by the user and *k* < *n*. The final division result must satisfy the following two conditions [22]: (1) each cluster must contain at least one data object; (2) the data set in each data object can and can only be contained in one cluster. When using a division-based clustering algorithm for partitioning data sets, the final clustering results are generated in a single step of execution rather than through multiple steps of joint execution. Multiple sets of clusters may be generated during the execution of the algorithm, but the final output result contains only one set of clusters. The method execution is terminated when the generated clustering sets satisfy the iteration termination condition, and in addition, a metric function is used to evaluate the degree of performance of the final clustering results.

Hierarchical-based clustering algorithms use hierarchical decomposition in the process of partitioning data objects in a data set. Cohesive hierarchical clustering algorithm is also known as bottom-up hierarchical clustering algorithm. First, each data object in the data set is considered as a cluster, and then each cluster is merged according to the merging rules, so that the number of data objects contained in the cluster becomes more, and the number of clusters becomes less, until the clustering result satisfies the set termination condition (e.g., the number of clusters satisfies a predetermined *k* value), or finally only one cluster remains [23].

The density-based clustering method divides the data set according to the distribution pattern of data objects in the data space, and the densely distributed data region in the data space is regarded as a cluster, while the main basis for the clustering division by the above two clustering methods is the distance between data objects. The main idea of this method is that if a fixed size region centered on a data object contains a certain number of data objects, the data objects are merged into the classification with the smallest distance between them, and the classification process continues until every data object in the data set is classified into a cluster. The above two clustering methods can only discover spherical clusters, while density-based clustering methods can not only discover spherical clusters, but also be used for mining other shape clusters. Density-based clustering algorithms include DBSCAN clustering algorithm [24] OPTICS clustering algorithm [25] and DENCLUE clustering algorithm [26].

The grid-based clustering algorithm divides the distribution space of data objects in a data set into a constant number of grid cells, which are combined together into a grid structure that represents the distribution of data objects in space, and the clustering operations during the execution of the algorithm are based on this grid structure [27]. This method has the advantage of fast clustering, because the time spent in processing the data depends on the division of the grid cells, and there is no relationship between it and the number of data objects. Grid-based clustering algorithms include STING clustering algorithm [28], CLIQUE clustering algorithm [29], and WAVE-CLUSTER clustering algorithm [30].

## 3. Algorithm Design

### 3.1. R-Tree Overview

**Figure 1:**
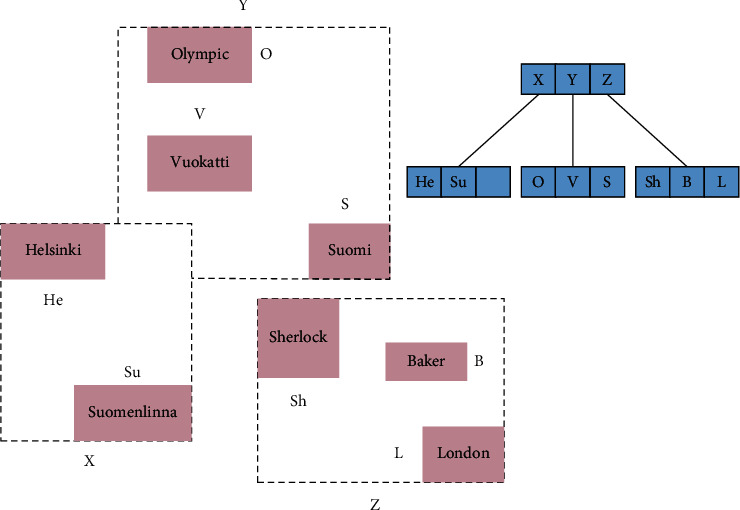
Example of *R*-tree index.

The R-tree index is an efficient spatial index with a structure similar to that of a *B*+ tree. R-tree adopts the spatial partitioning idea of a *B*-tree and uses the method of decomposing and merging nodes for deletion and addition to ensure the balance of the tree. For an *R*-tree of order *M*, each non-leaf node in the R-tree is composed of several (*P*, MBR) data pairs. The Minimal Boundary Rectangle (MBR) is the smallest bounding rectangle containing its corresponding children. MBR is a broad concept, which is a rectangle in two dimensions, but it is a rectangle with Minimum Bounding Volume (MBV) in three dimensions, and so on, up to higher dimensional spaces. *P* is a pointer to its corresponding child node. Each leaf node in the R-tree consists of a number of OI and MBR, and MBR is the smallest outer rectangle containing the corresponding spatial object. Oi is the identifier of the spatial object, by which the details of the corresponding spatial object can be obtained.  Figure 1 shows an example of R-tree index.

The construction of an R-tree requires the following properties to be satisfied: (1) there are *m* to *M* record indices (or entries) contained in the leaf nodes of the non-root nodes, while the number contained in the leaf nodes of the root node can be less than *m,* where *m*=*M*/2 . (2) There are records (entries) in all leaf and non-leaf nodes, and *i* is the smallest rectangle that can completely cover the points represented by these entries in space. (3) Each non-leaf node other than the root node has from *m* to *M* child nodes. (4) All leaf nodes are located at the same level, so the R-tree is a balanced tree.

Currently, R-tree can be divided into two parts according to how it is constructed: dynamic indexes and static indexes. Dynamic indexes are constructed at runtime for dynamic data. The high rectangular overlap of traditional *R*-tree leads to degraded query performance of the index and does not represent the current data effectively. The introduction of various node splitting and reinsertion methods provides a solution to differentiate the dynamic variables of R-tree. For example, *R∗*-tree and *R* + -tree improve the *R*-tree using splitting and forced insertion algorithms to optimize the coverage of closed rectangles in internal nodes for better tree structure and improved retrieval performance. However, R-tree and its variants insert data from the empty tree, so it leads to large insertion cost; only local optimization rather than global optimization is performed when inserting data, and there are many overlaps between the constructed rectangles, and the tree structure is unreasonable.

Using dynamic insertion algorithms to build indexes can provide a lot of dead space in the nodes, resulting in performance degradation. Moreover, R-tree variants do not utilize the known dataset during insertion. If the input spatial data is packed heuristically, and the R-tree is built statically, the space utilization is improved. Therefore, for static spatial data with little change in the data and no frequent deletion and update operations, a method of sorting MBRs is proposed to construct the R-tree. The R-tree index is constructed by sorting MBRs in a two-dimensional space according to certain rules. The leaves of the tree are first populated, and then the rest of the index is gradually constructed in a bottom-up manner. This construction method reduces the overlap rate and construction complexity among nodes, but it is not particularly effective because of the high resource consumption and consumption in the preprocessing stage. The TGS-R tree constructs the R-tree in a top-down manner by recursively splitting the dataset into two subsets. This method minimizes the objective function cost on the MBRs of each split subset, and each subset has a certain number of rectangles and performs significantly better than STR tree on point and range queries. Another construction method is to optimize the R-tree with cache, similar to cache-conscious *B*+ trees.

### 3.2. R-Tree Clustering Model-Based Literature Information Retrieval

In order to achieve effective access to large-scale data, this paper uses a clustering model based on R-tree indexing for data retrieval of scientific and technical literature. When constructing the R-tree clustering model, if the distribution law of data is unknown, presetting the clustering centers will make the final clustering results deviate from reality, thus affecting the efficiency of the constructed R-tree model index. In order to determine the clustering centers effectively, the DCC algorithm is introduced in this paper to construct the R-tree model. Let the distance index of measuring neighboring objects be *R*, which is denoted as

(1) $$\begin{array}{c} R=\frac{1}{\sqrt{m/D}}, \end{array}$$

where *m* is the amount of spatial data, *D* is the range of the given spatial region, and *d*_*i*_ is the distance from the data to *i*. If *d*_*i*_ < *R*, mark *i* as a neighboring object of the data; if *d*_*i*_ > *R*, mark it as a nonneighboring object of the data.

Given that *r*_1_, *r*_2_,…, *r*_*m*_ is the set of *R*^*d*^ space data, let *c*_*l*_ be the clustering center of *l*, and then the distance (distance function) between *r*_*i*_ and *c*_*l*_ is

(2) $$\begin{array}{c} d\left(r_{i},c_{l}\right)=\sqrt{{\left(r_{i}^{1}-c_{l}^{1}\right)}^{2}+{\left(r_{i}^{2}-c_{l}^{2}\right)}^{2}+\cdots +{\left(r_{i}^{d}-c_{l}^{d}\right)}^{2}}. \end{array}$$

Let the sample of the lth class be *S*_*l*_={*r*_*l*1_, *r*_*l*2_,…, *r*_ln_}; that is, it contains *n* data, and then the mean point of the class is *c*_*l*_=(*c*_*l*_^1^,…, *c*_*l*_^*k*^,…, *c*_*l*_^*d*^), where *c*_*l*_^*k*^ is the kth attribute of *c*_*l*_, which can be expressed as

(3) $$\begin{array}{c} c_{l}^{k}=\frac{r_{l1}^{k}+r_{l2}^{k}+\cdots +r_{\ln}^{k}}{n}. \end{array}$$

When selecting the clustering center, first, obtain the mean point *c*_*l*_ of the data in the class, and then calculate the distance between this mean point and other data, obtain the neighboring objects of *c*_*l*_ according to the distance index *R*, calculate the mean point *c*_*l*_′ of the neighboring objects, and select the spatial data closest to *c*_*l*_′ as the clustering center, labeled as *r*=argmin(*d*(*c*_1_′, *r*)), where *r*={*r*_*l*1_, *r*_*l*2_,…, *r*_*ln*_}.

**Figure 2:**
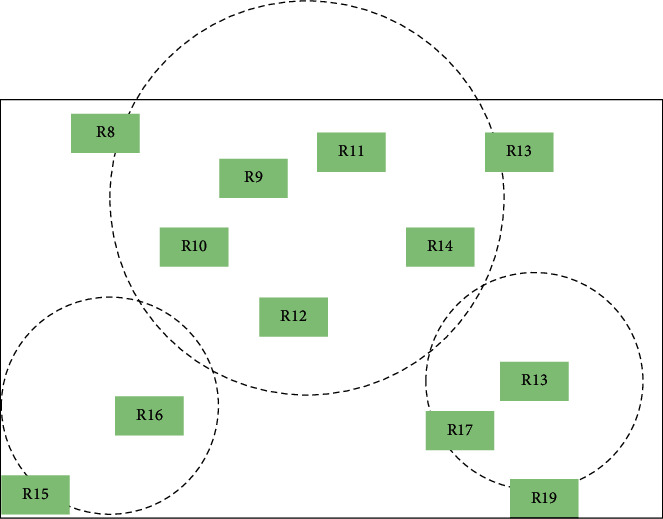
Three clustering centers.

**Figure 3:**
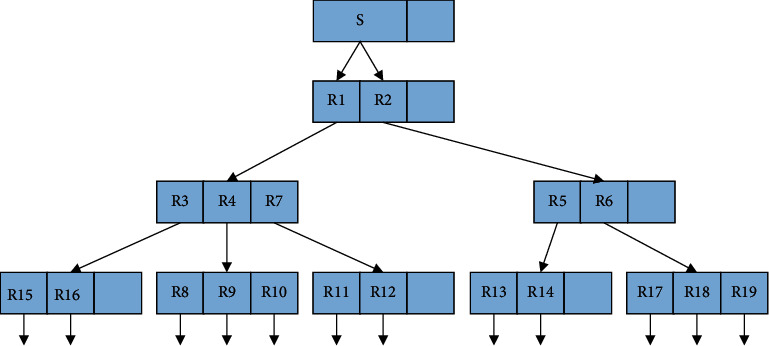
Hierarchy and its R-tree model.

The R-tree generation for any spatial data set is shown in Figures 2 and 3. The base rectangles are grouped according to the dynamic determination of clustering centers DCC algorithm. For example, in Figure 2, *R*12, which is the closest to the mean, is selected as the initial cluster center, and *k* = 1. Then, *R*19, which is the farthest from R12, and R8, which is the farthest from *R*19, are selected as the cluster centers, and the clustering is started. *R*13, *R*14, *R*17, and *R*18 are divided into *R*19, *R*9, *R*10, *R*11, *R*12, *R*15 and *R*16 are divided into *R*8, at which point two clusters are formed *k* = 2, and the cluster centers and cluster measurement functions are calculated. The cluster with the largest radius and its cluster center R12 are selected from the two clusters, and *R*15, which is farthest from R12, and R11 and R18, which are farthest from *R*15, are selected as cluster centers and reclustered, and then their cluster centers and cluster measurement functions are calculated, and *k* = 3. The cycle is repeated, and the value of *k* keeps increasing until the clustering function converges.

The R-tree index itself has complete index creation (insertion), query, and node deletion algorithms, querying geometric data in the database based on R-tree index. By building R-tree indexes in the database, the efficiency of multiuser data retrieval can be greatly improved.

## 4. Experiments

### 4.1. Experiment Preparation

**Figure 4:**
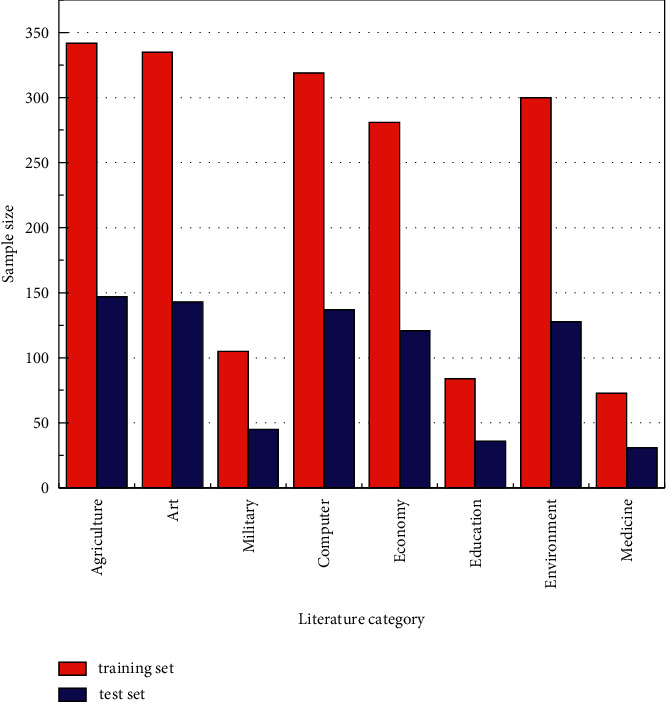
Dataset division.

Before the experiment, considering the calculation process, the text similarity is larger, and the dimensionality generated is higher, and the text category, title, keyword, and keyword are analyzed when clustering is performed to reduce the dimensionality generated by the text during the calculation, while the experiment is conducted using the method proposed in this paper; the experimental environment is as follows: the system environment used for the experiment is windows 10 64 bit, the processor is Inter i5 5200 2.2 G, and the memory is 6G. The dataset is derived from the text classification language library provided by the natural language processing group of a university database center, the total number of documents is 19637, with 20 categories, among which eight categories of agriculture, art, military, computer, economy, education, environment, and medicine are selected, with a total of 2627 texts, of which the total number of texts used for training is 1839, and the remaining 788 documents are used for testing, and the experimental data are shown in Figure 4. For the evaluation of document clustering effect, the commonly used index parameters are the document checking accuracy P (precision), checking completeness *R* (recall), and F1-score (correlated with accuracy and checking completeness).

### 4.2. Analysis of Retrieval Efficiency Based on R-Tree Clustering

**Figure 5:**
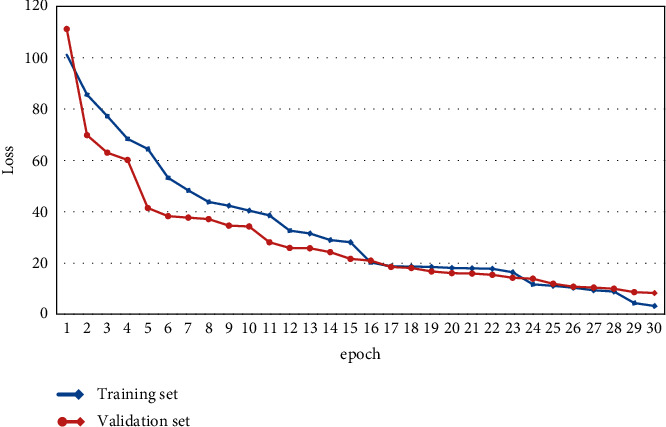
Lossy convergence diagram of the training process.

**Figure 6:**
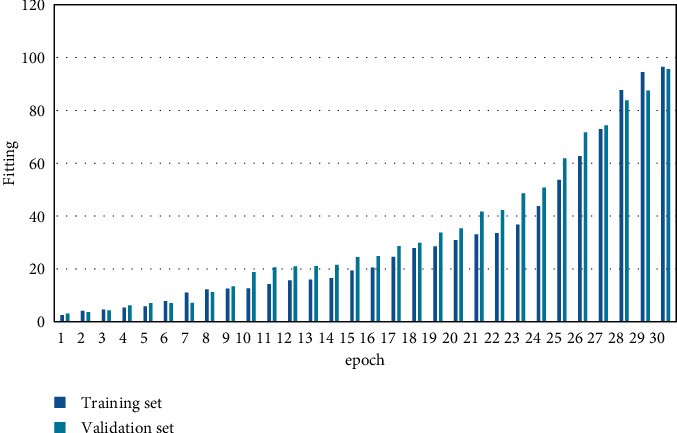
Performance improvement of the training process.

The size of R-tree is determined by the amount of data. Set *W* as the network bandwidth resource and *T* as the amount of data. Use N to denote the number of queries and *Q* to denote the query complexity. The experimental variable *α* denotes the available bandwidth, and *β* denotes the number of tasks. The parameters are set as shown in Table 1. The loss convergence and performance improvement of the training process are shown in Figures 5 and 6.

**Figure 7:**
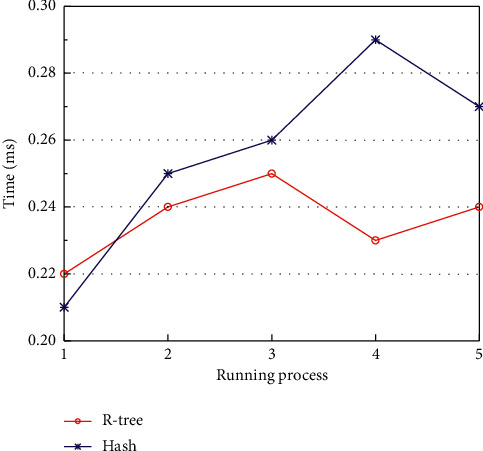
System performance at. *α*=1,  *β*=0.

The experimental results in Figure 7 show that when the task size is small, the retrieval time of the system deploying R-tree is slower than hash index at the beginning, but after a period of time, the speed of the system improves significantly. The reason for this result is that the system deploying R-tree has to build R-tree at the beginning of retrieval, and the time complexity of R-tree constructed by using the method in this paper is *O*(*n*^*k*^ × *t*), where *k* is the number of clusters, *t* is the number of iterations, and *n* denotes the amount of data. And the time complexity of hash index is *O*(1). So, at the point of system operation, R-tree index is slower than hash index. However, the R-tree constructed in this paper can effectively reduce the overlap and coverage between MBRs, making the generated tree structure compact and with fewer multipath queries, and improving the retrieval efficiency. After running the system for a period of time, the retrieval efficiency of R-tree is significantly higher than that of hash index.

**Figure 8:**
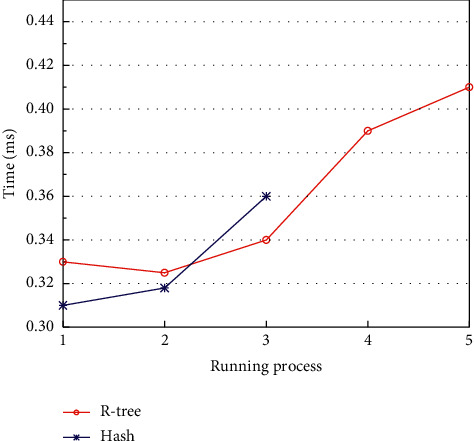
System performance at. *α*=0,  *β*=1.

As shown in Figure 8, the system performance of deploying R-tree outperforms hashing when the task volume is high. The advantage is especially obvious in the state of busy network. When the network is busy, the system deploying hash index crashes and fails to operate after several experiments, making it impossible to perform subsequent experiments. But the system deploying R-tree can still perform the subsequent operations. This is because, for R-tree, the more the lookups are, the more the data it contains, and the more the search paths are, thus affecting the query response time. And the creation and maintenance of the hash table put a great burden on the computational performance of the computer, so when the amount of data is large, it will have an adverse effect on the system performance after running for a period of time.

### 4.3. Search Precision Analysis

In this paper, we compared it with KNN and DBSCAN algorithms. The classification comparison of the three algorithms at different values is shown in Table 2. From the experimental data in the table, it can be concluded that the KNN algorithm has the best clustering effect when the values of P, *R*, and F1 reach the maximum when the value is 19; the DBSCAN algorithm reaches the maximum value when the value is 14; the algorithm in this paper has the best clustering effect when the values of P, *R*, and Fl are maximum when the value is 17.

**Figure 9:**
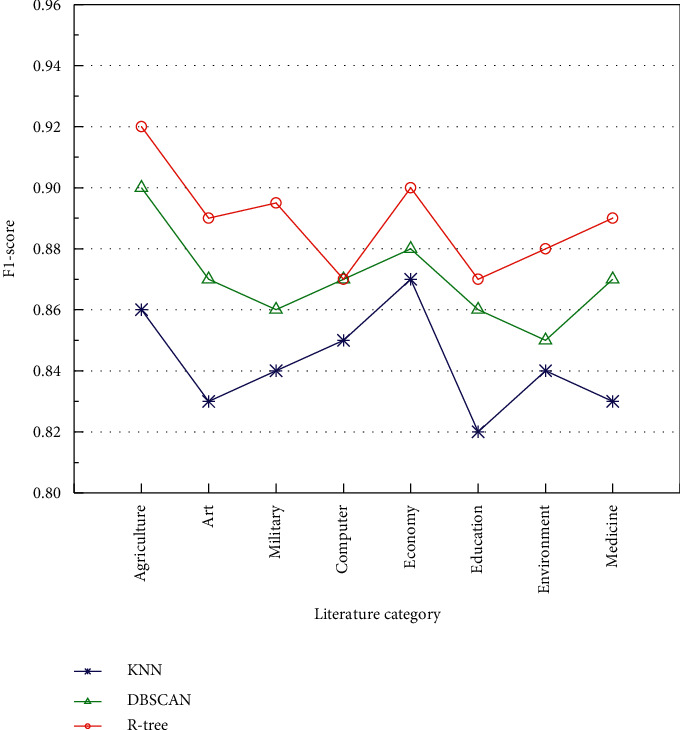
Comparison of F1-score of three algorithms.

On the premise of obtaining the optimal values, the second stage of the experiment was conducted to compare the superiority of the three different algorithms during the experiment, and the experimental data are shown in Figure 9 and Table 3. The comparison of the F1 values and time consumption of the KNN algorithm and the DBSCAN algorithm with the method proposed in this paper after clustering the data set can be seen from the two sets of data in Figure 9 and Table 3. In Figure 9, the DBSCAN algorithm shows a significant improvement in the F1 values obtained relative to KNN, while the algorithm in this paper has further improved on top of the first two algorithms. In Table 3, the KNN algorithm takes the highest time in clustering, and DBSCAN takes 18 seconds more time relative to the algorithm proposed in this paper.

## 5. Conclusions

Compared with traditional retrieval systems, the advantage of scientific and technical literature retrieval systems lies in their ability to process semantic information, discover potential knowledge from unstructured text, realize knowledge retrieval, and meet higher retrieval needs of users. By studying and analyzing the existing semantic retrieval systems for scientific and technical literature, we can find that the semanticization degree of the system depends on the depth of semantic mining of the literature, and the mining and discovery of specified information can be achieved to a large extent by using existing technologies such as text mining, natural language processing, and cluster analysis. In this paper, firstly, from the current situation of scientific and technical literature query service in the context of big data, we construct a literature query model in the context of big data by combining the clustering analysis technology and lay a theoretical foundation for the design of clustering search engine. Specifically, this paper proposes a new search method for the characteristics of diverse types of literature data and large data volume, combined with R-tree indexing. Experimental results show that the method of this paper greatly improves the efficiency and accuracy when retrieving large amount of scientific and technical documents. In the future, we plan to conduct research on document clustering algorithms using recurrent neural networks for a large number of scientific and technical document query services.

## Figures and Tables

**Table 1:** Parameter settings.

Parameters	Values
*W*	100 MB
*T*_1_, *T*_2_	0.47 MB, 4.48 mb
*N*_1_, *N*_2_	5549, 12068
*Q*_1_, *Q*_2_	1, 4
*α*=0	100 MB
*α*=1	50 MB
*β*=0	*T*_1_+*N*_1_+*Q*_1_
*β*=1	*T*_2_+*N*_2_+*Q*_2_

**Table 2:** Comparison of retrieval accuracy with other algorithms.

Times	KNN				DBSCAN				Proposed			
	Values	*P*	R	F1	Values	*P*	R	F1	Values	*P*	R	F1
1	7	72.9	73.1	73.0	8	85.6	80.0	82.7	9	81.3	84.5	82.9
2	10	74.4	72.6	73.5	10	82.9	91.1	81.9	11	83.2	82.1	82.6
3	13	77.4	74.2	75.8	12	78.6	91.2	84.4	13	85.6	88.3	86.9
4	16	79.7	78.6	79.1	14	95.2	95.6	95.4	15	92.3	95.7	94.4
5	19	82.7	79.4	81.0	16	91.4	93.2	92.3	17	95.8	96.2	96.0
6	22	79.4	76.6	78.0	18	85.3	90.2	87.7	19	86.8	91.4	89.0
7	25	78.3	73.3	75.7	20	83.6	87.1	85.3	21	83.5	87.3	85.4
8	28	76.5	72.4	74.4	22	81.3	84.6	82.9	23	81.1	83.2	82.1

Mean		77.7	75.0	76.3		85.5	87.9	86.6		86.3	88.6	87.4

**Table 3:** Time consumed by the three algorithms to query all literature.

Method	Time (s)
KNN	1045
DBSCAN	893
Proposed	875

## Data Availability

The datasets used during the current study are available from the corresponding author upon reasonable request.
